# The psychological impact of first-time childbirth on parents

**DOI:** 10.1371/journal.pone.0334669

**Published:** 2026-03-24

**Authors:** Bo Zhu, Yani Guo, Meng Du, Xiangjun Zhou

**Affiliations:** 1 Wenzhou People’s Hospital Affiliated to Hangzhou Medical College, Wenzhou, Zhejiang, China; 2 The Wenzhou Third Clinical Institute AffFiliated To Wenzhou Medical University, Wenzhou, Zhejiang, China; 3 School of Management Science and Engineering, Shanxi University of Finance and Economics, Taiyuan, Shanxi, China; 4 Department of Finance, Shandong Technology and Business University, Yantai, Shandong, China; 5 School of Business, Wenzhou University, Wenzhou, Zhejiang, China; Public Library of Science, UNITED KINGDOM OF GREAT BRITAIN AND NORTHERN IRELAND

## Abstract

The psychological effects of first-time childbirth on parents have long been a focal point in social science research. This study provides a new perspective by applying demographic transition theory to explore how the experience of having a first child influences parents’ mental health. Our findings indicate that the arrival of a first child not only increases family size but also brings about significant psychological and emotional adjustments in parents. These changes are often driven by pressures associated with financial stability, career development, and shifts in personal identity, which prompt parents to reconsider their life goals and values. Moreover, the psychological effects of first-time childbirth vary notably across different socioeconomic backgrounds. This paper offers empirical insights that can guide policymakers and social organizations in developing targeted support and intervention strategies to enhance parental well-being during the transition to parenthood.

## Introduction

With the continuous decline of fertility rates in China, issues related to family reproduction have become a focal point of social concern. Despite the implementation of the two-child policy in 2016 and its relaxation to a three-child policy in 2021, China’s fertility rate dropped below 1.1 in 2022, significantly lower than the generational replacement level of 2.1 [1]. This demographic trend has led to accelerated challenges such as aging, declining birth rates, and an increase in the number of unmarried individuals.

From the perspective of population transition theory, having a first child is a significant life event that brings both joy and new challenges for parents. The economic pressures associated with raising a child [2],combined with societal and cultural shifts, can impact parents’ psychological well-being [3]. As educational opportunities for women increase and their roles in the workplace expand, many families choose to delay childbirth or have fewer children, which further complicates the balance between work and family roles [4]. Moreover, becoming parents can lead to a reevaluation of personal goals and values, contributing to identity shifts and psychological stress [2].

The differentiated impact of first-time childbirth on the mental health of fathers and mothers is an area that has received limited attention [2]. Understanding the heterogeneous effects of first-time childbirth is crucial for developing effective support measures and interventions to enhance parental mental well-being [5].

This study aims to explore the impact of first-time childbirth on parental mental health, with the goal of improving parenting quality and positively influencing future reproductive decisions. Using both quantitative and qualitative methods, we aim to better understand how to help parents transition smoothly into their new roles and enhance their mental well-being.

To ensure the accuracy and reliability of our research, we utilized data from the China Family Panel Studies (CFPS) from 2010 to 2020. We adopted the first childbirth rate in the same village as an instrumental variable for first-time childbirth to alleviate potential endogeneity problems. With this approach, we aim to provide a more accurate estimate of the impact of first-time childbirth on parents’ psychological health.

The structure of the remaining content of this paper is as follows: The second section focuses on theoretical analysis and research hypotheses; the third section discusses research design; the fourth section presents estimation results and analysis; the fifth section delves into extended analyses; and the final section offers conclusions and policy recommendations.

## Literature review and theoretical analysis

### Literature review

The issue of childbirth and its impact on parents’ psychological health has garnered attention from numerous scholars [6]. The literature in this domain can be categorized into two primary dimensions: the direct effects of childbirth on parents’ mental well-being and the influence of external factors such as societal, cultural, and economic contexts on this relationship.

(1) Direct impacts of childbirth on parents’ psychological health.

Numerous studies have focused on the direct effects of childbirth on parents’ mental health. For instance, some research indicates that parents might experience a surge in happiness during the initial period after a child’s birth [7]. However, as the child grows, this feeling may gradually diminish [8,9]. In specific scenarios, like the arrival of a newborn, parents might exhibit temporary symptoms of depression [10,11].Furthermore, for mothers with multiple births, their childbirth experiences might have a more significant impact on their psychological well-being [12,13]. For those becoming parents for the first time, their experiences might differ from parents with prior child-rearing experiences [14,15]. The societal, cultural, and economic backdrop plays a pivotal role in shaping the relationship between childbirth and parents’ psychological health.

While numerous studies have delved into the direct impact of childbirth on mental well-being, the influence of external factors remains relatively under-researched. Racine, Nicole, et al. (2019) [16] examined how societal support mitigates the psychological stress arising from childbirth. Additionally, cultural perceptions about childbirth, which vary across different backgrounds, also significantly influence parents’ mental states. Economic conditions have also been proven to be a vital factor affecting this relationship [17,18]. Especially during the critical moment of childbirth, certain external factors can either amplify or mitigate its impact on parents’ psychological health.

(2) The existing literature provides in-depth insights into the relationship between childbirth and parents’ psychological well-being.

However, most current studies focus primarily on overall parental mental health of parents during pregnancy and the neonatal period, often overlooking the unique context of first-time childbirth [19,20]. The psychological, physiological, and societal changes arising from the birth of the first child may differ significantly from those experienced during subsequent childbirths. First-time parents might face more significant lifestyle changes, heightened financial pressures, and a redefinition of roles with their partners [21,22]. Given the backdrop of an aging population, declining birth rates, and increasing instances of people choosing to remain unmarried, studying the transition from no child to one child becomes particularly crucial and unique in the Chinese context. This transition is not just about changes in family structure [23, 24] but also pertains to the establishment and recognition of parental identity [25]. This identity shift could have long-term implications on parents’ psychological health [26], quality of life, and future family planning decisions [27,28].

This paper aims to deepen the understanding of the relationship between childbirth and parents’ psychological health, exploring the channels through which first-time childbirth impacts parents’ mental well-being. Firstly, within the framework of demographic transition theory, this study analyzes the effects of first-time childbirth on parents’ psychological health, measuring it in terms of happiness and depressive symptoms, thereby providing precise data support for relevant policy formulation. Furthermore, by comparing the psychological changes in fathers and mothers after childbirth, we aim to uncover which factors, beyond gender, contribute to the gender differences in the relationship between childbirth and mental health. Identifying these potential influencing factors will further complement and enhance the knowledge base in this field. Lastly, while most related studies utilize cross-sectional data or a mix of multiple periods, lacking observations of the same individual over multiple periods, this paper employs panel data to eliminate biases caused by unobservable individual fixed effects.

### Theoretical analysis and research hypotheses

The theory of demographic transition refers to the historical shift from high birth rates and high death rates in societies with lagging technological, educational, and economic development to low birth rates and high death rates in societies with advanced technological, educational, and economic development [29]. The negative correlation between fertility rates and industrial development has become one of the most widely accepted findings in social science [30]. This theory comprises a five-stage model of demographic transition [31]. Stage One: High birth and death rates due to poor medical conditions, prevalent diseases, and the absence of modern medical technology. High birth rates are attributed to the lack of contraception, with children serving as a critical source of labor and high child mortality rates. In this stage, both death and birth rates are high, keeping the overall population relatively stable. Stage Two: High birth rates and declining death rates. Improvements in medical conditions, better nutrition, and advancements in public health and sanitation lead to a decline in death rates. Despite this, birth rates remain high, resulting in rapid population growth. Stage Three: Declining birth and death rates. As the economy develops, opportunities for women’s education increase, leading to smaller family sizes. Contraception becomes more widespread and accepted. Birth rates begin to decline, and as death rates continue to decrease, population growth starts to slow. Stage Four: Low birth and death rates. Family sizes reduce further, women’s societal and economic status improves, and contraception becomes ubiquitous. Both birth and death rates are low, leading to slow or stable population growth. Stage Five: Extremely low birth rates and low death rates. Socio-economic pressures result in even smaller family sizes and reduced fertility desires. Birth rates decline further, falling below death rates, leading to a decreasing population.

This framework offers a macro perspective, helping us understand how economic, societal, cultural, and health factors collectively influence population structure and growth. In studying the relationship between first-time childbirth and parents’ psychological well-being, it becomes pertinent to consider how these macro factors interact with micro-level family decisions. Broadly speaking, in the demographic transition theory, external factors impact parents through channels like physical health, job income and opportunities, and confidence in the future.

#### Parents’ physical health.

First-time childbirth can have a multifaceted impact on parents’ physical health. For instance, delayed childbirth may increase health risks for mothers [32]. Additionally, an unequal distribution of health resources within the family, such as one parent receiving less medical attention or fewer opportunities for rest and recovery compared to the other, might influence the overall health conditions of both parents [33]. This could be exemplified by a scenario where the primary caregiver, often the mother, neglects her own health needs in favor of focusing on the child, thereby exacerbating her risk of postpartum complications [34]. Moreover, becoming parents for the first time may lead to changes in lifestyle, such as reduced exercise and irregular diet [35].Research indicates that new mothers might face higher risks of chronic diseases like diabetes and hypertension post-delivery. These physical health issues not only directly impact parents’ psychological well-being [36] but might also indirectly affect their mental health by influencing the family’s economic condition, partner relationships, among other factors [37,38]. It’s evident that first-time childbirth might have both direct and indirect effects on parents’ physical health.

Research Hypothesis 1: First-time childbirth may lead to a decline in parents’ physical health, further affecting their sense of happiness and increasing the risk of depressive symptoms.

#### Job income and employment opportunities.

First-time childbirth can have significant economic implications, affecting parents’ work income and job opportunities. For instance, parents might opt to delay childbirth to first pursue their career goals, which could impact their work income and opportunities. Simultaneously, the pressures of balancing work and parenting roles might affect their job performance and income. Parents, especially mothers, might take temporary breaks from their careers due to maternity leave or breastfeeding leave, potentially affecting their job opportunities and career progression [39,40]. Changes in work and economic status might directly influence parents’ psychological health [41–43]. Hence, first-time childbirth might have long-term repercussions on parents’ career trajectories and financial conditions.

Research Hypothesis 2: First-time childbirth can lead to a decline in parents’ work income and potentially impact their career advancement opportunities, further possibly reducing their sense of happiness and elevating the risk of depressive symptoms.

#### Confidence in the future.

First-time childbirth may not only alter parents’ expectations for the future but also impact their confidence in what lies ahead. This change could be driven by various factors, including cultural values, societal norms, and personal experiences. On one hand, the arrival of a child may instill more hope and optimism in the family [44]. On the other hand, facing the challenges and pressures of parenting might lead parents to feel more uncertain and apprehensive about the future. Such changes in expectations and confidence about the future can profoundly affect parents’ mental health [8]. With the child’s arrival, the focus often shifts towards providing the best possible educational opportunities, with parents harboring higher aspirations for their child. This shift can involve efforts to enroll the child in superior schools and secure better educational resources. However, the daunting costs of education combined with intense competitive pressures might generate concerns about their ability to provide these opportunities. Concerns are not only financial; health issues and related medical expenses can also heighten apprehensions about future well-being. As a result, parents may prioritize savings for their child’s education and healthcare needs, although the financial burden of raising a child and prevailing economic uncertainties could erode their confidence in maintaining financial stability [45]. Furthermore, becoming parents often entails adapting to shifts in cultural and societal values, which might influence their expectations and redefine their measures of success. Societal definitions of “success” continue to shape parents’ aspirations for their child’s future.

Research Hypothesis 3: First-time childbirth can lead to changes in parents’ expectations and confidence in the future, especially concerning education, health, and economic security. Such changes might further influence their sense of happiness and risk of depressive symptoms.

## Research design

### Data source

This study relies on the micro-database of the “China Family Panel Studies” (CFPS). The CFPS is a stratified multi-stage sampling survey that covers 25 provinces (cities, autonomous regions) of mainland China, excluding Xinjiang, Tibet, Inner Mongolia, Ningxia, and Qinghai, representing 94.5% of the mainland Chinese population. This database encompasses a wealth of individual-level information, including socio-economic, demographic, and a range of subjective variables. Notably, the survey for adults includes a depression scale to gauge mental health status, encompassing aspects like depressive symptoms, emotions, and interpersonal relationships. Furthermore, it also incorporates economic data, such as family income and consumption expenditure. The CFPS has been conducted since 2010 and has completed six rounds to date, specifically in 2010, 2012, 2014, 2016, 2018, and 2020. For the purposes of this research, we utilized data from all six rounds spanning 2010–2020. As the United Nations’ population forecast typically defines women of reproductive age as being between 15 and 49 years old, and considering the legal marriage age in China is not earlier than 20 years, we limited the age range of household heads to between 20 and 49 years for data processing. To mitigate the impact of outliers on the results, we adjusted these variables at the 1% and 99% percentiles. After filtering and processing, we obtained an unbalanced panel data spanning six survey rounds, encompassing 119,011 samples.

### Variable selection

#### Dependent variables.

Depression: This variable primarily originates from the psychological health measurements in the CFPS questionnaire. The 2010 and 2014 surveys employed the Kessler Psychological Distress Scale (K6), whereas the 2012, 2016, 2018, and 2020 surveys utilized the Center for Epidemiologic Studies Depression Scale (CES-D). This study referred to various CFPS reports from 2010, 2012, and 2016 to construct these indicators. To ensure consistency, based on the CFPS technical report, we standardized all questions to reverse scoring and summed up the scores for each question. Furthermore, to avoid dimensional differences and facilitate coefficient interpretation, we standardized the total score to have a mean of 0 and a standard deviation of 1, yielding a psychological health indicator. A higher score indicates a poorer mental health state. As the CES-D scale contains reverse-worded questions, we reversed the scores for these questions to ensure that a higher score for each question indicates a more severe degree of depression. This ensures that the total score points towards a unified mental health indicator. Additionally, the self-rating standards of the K6 and CES-D scales differ. To standardize this, we converted the 2010 and 2014 data to reverse scoring.

Happiness: Happiness is a complex emotional experience influenced by various factors. It’s not solely about material prosperity but more related to psychological and emotional satisfaction. Residents’ subjective well-being can be understood as an evaluation based on one’s standards, comparing their actual life state with their ideal life condition. It’s a subjective sensation of “seeking benefits and avoiding harms,” i.e., people pursue factors that bring happiness and avoid those that might lead to unhappiness. In the CFPS database, the original questionnaire included a subjective question about happiness: “How happy do you feel?” This question effectively captures an individual’s subjective well-being since it allows respondents to evaluate based on their life experiences and emotional state. To quantify this experience, the answer range is set from 0 to 10, where 0 means “least happy” and 10 means “happiest.” This design ensures that respondents express their happiness on a continuous scale, more accurately reflecting their subjective experience. A higher score indicates stronger happiness.

#### Explanatory variables.

First-time Childbirth: Although the CFPS database does not provide detailed records on the fertility history of married women, we can obtain complete and symmetrical parent-child and couple data through family relationship matching. By matching these relationships, we identified the number of children each couple had at each survey wave and calculated the age of their oldest child. By subtracting the child’s age from the survey year, we inferred the baseline year of first childbirth for each household, denoted as T = 0, for the couple. This baseline year represents the year when the couple had their first child. To calculate the time from the first childbirth for the couple at each survey point, one simply needs to subtract the baseline year from the calendar year of each survey point. This provides us data for each year since the first childbirth.

#### Mechanism variables.

Physical Health: Changes in parents’ physical health post first-time childbirth are a significant channel influencing their psychological well-being. In the CFPS database, a question asks whether the respondent had been diagnosed with a chronic illness by a doctor in the past six months. This metric reflects potential impacts on physical health, where a response of 1 denotes “yes” and 0 denotes “no.”

Job Opportunities: After the first childbirth, many parents face adjustments and changes in their careers. Consequently, we used a question about satisfaction with the promotion opportunities in the current job: “How satisfied are you with the promotion opportunities in this job?” This captures the subjective feelings about job advancement opportunities post first-time childbirth, with answer choices ranging from 1 (very unsatisfied) to 5 (very satisfied).

Job Income: First-time childbirth may impact the family’s economic condition and individual’s satisfaction with income. We employed a question about job income satisfaction: “How satisfied are you with the income from this job?” The answer choices range from 1 (very unsatisfied) to 5 (very satisfied).

Confidence in the Future: For many, first-time childbirth signifies a fresh beginning, which may influence their outlook and confidence in the future. To measure this impact, this study utilizes the question: “How confident are you in your future?” where 1 denotes “not confident at all” and 5 indicates “very confident.”

#### Control variables.

To analyze the impact of first-time childbirth, it’s imperative to ensure that other potential confounding factors are controlled. This paper incorporates a series of control variables that might influence the psychological well-being of parents after first-time childbirth.

Firstly, we controlled for basic demographic features. This includes age (reflecting that age at the time of childbirth might influence the experience post first-time childbirth), gender (as men and women might have different post-childbirth experiences), years of education (which can elucidate how educational level influences perspectives on first-time childbirth), household registration status (considering urban-rural differences and related living conditions), and Communist Party membership status. Secondly, we also controlled for family-related variables. This encompasses family financial situation (a crucial metric gauging the economic status of the family), marital satisfaction, spouse’s satisfaction with household chores, and child-rearing expenses which can greatly influence the decision to have a child. To further precisely control potential confounding factors, this study incorporated individual fixed effects to capture impacts of individual characteristics that remain constant over time. Simultaneously, we controlled for fixed effects such as village (cid) and county (countyid) to consider varying cultural, economic, and policy backgrounds across regions. Lastly, to control for time trends and the impact of specific years, we included time fixed effects in our model.

### Descriptive statistics

As shown in Table 1, the full-sample descriptive statistics for key variables include only the dependent variables and core explanatory variables. Over the past decade, both the fertility patterns of Chinese families and the psychological well-being of parents have undergone significant changes. An analysis of Table 1 reveals the following findings: The change in the “Firstchild” variable indicates that the proportion of families experiencing first-time childbirth increased from 35.23% in 2010 to 45.59% in 2012, possibly reflecting a stronger fertility desire under the socio-economic conditions of the time. However, by 2014, this proportion declined to 38.87%, fluctuating in the subsequent years until stabilizing at 37.03% in 2020. This trend may suggest multiple factors behind family fertility decisions, such as pressures from economic slowdown, distribution of educational resources (i.e., how educational opportunities and facilities are allocated across different regions and social groups), shifts in cultural expectations, and the impact of the pandemic.

**Table 1:** Summary statistics for key variables.

	Pooled years		2010	2012	2014	2016	2018	2020
	Mean	Std.dev.	Mean	Mean	Mean	Mean	Mean	Mean
Firstchild	0.3782	0.5	0.3523	0.4559	0.3887	0.3578	0.338	0.3703
Happiness	7.0297	4	5.7588	7.8665	7.5501	7.7034	7.4733	7.4600
Depression	−0.0293	0.9	−0.0395	−0.0782	−0.0636	−0.0409	−0.0075	0.0952
Confident	4.003	1.0	3.8371	3.8264	4.1467	4.0055	4.1589	4.1214
Observations	119011		19987	20503	23444	21210	19091	14776

The “Happiness” index also underwent notable fluctuations during this decade. It rose from 5.7588 in 2010 to 7.7034 in 2016 and then slightly decreased to 7.4600 in 2020. This rise in happiness might be associated with the nation’s economic growth, improvements in living standards, and changes in social policies. However, the slight decline in 2020 might hint that, although the overall happiness of residents remains relatively high, there are emerging economic and social pressures and challenges.

Concurrently, the “Depression” index reveals that the psychological health of respondents hasn’t been consistently stable. While the average value of depression symptoms slightly decreased from 2010 to 2012, the depression index increased annually up to 2020, reaching a value of 0.0952. This could be attributed to pressures brought about by economic deceleration and the health-related stresses induced by the 2019 COVID-19 pandemic.

The “Confident” variable indicates that respondents’ confidence showed minor fluctuations between 2010 and 2020 but, overall, displayed an upward trend. This might be associated with increased educational opportunities, possibilities of career advancement, and socio-cultural transformations (i.e., changes in the values, norms, and practices of a society that affect its structure and the lifestyles of its members).

From the aforementioned indicators, it’s evident that the fertility patterns of Chinese families and the psychological well-being of parents have undergone significant changes over the past decade. These alterations might have been impacted by macroeconomic factors, policies, socio-cultural influences, as well as internal family decisions, beliefs, and health aspects.

**Fig 1:**
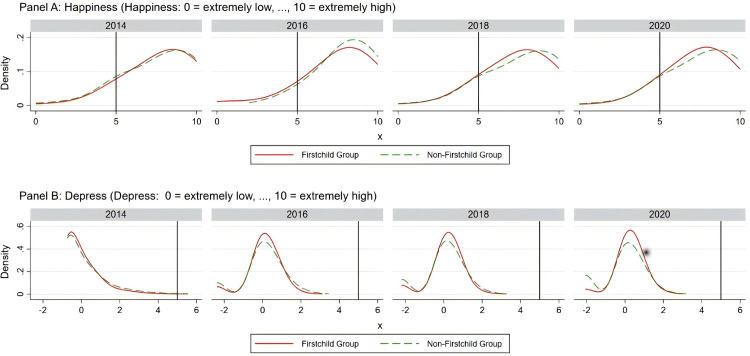
Distribution of Happiness and Depression.

As illustrated in Fig 1, there is a distinct distribution difference between parents who experienced first-time childbirth and those who did not across two major psychological health indicators—“Happiness” and “Depress”. The data in Panel A of Fig 1 shows that parents who had their first child had a much higher proportion of happiness scores below 5 compared to parents who did not have their first child in consecutive observation years. This difference was stable across different years, suggesting it might be a long-term characteristic. In this context, “parents who did not have their first child in consecutive observation years” refers to those who either had subsequent children in later years or did not have any more children after the first one. Therefore, parents who had their first child in 2014 would be part of the “first child” group for that year but would be considered in the “non-first child” group in subsequent years (i.e., in 2016, 2018, and 2020). Panel B displays the annual distribution for depression scores, showing that parents who had their first child generally scored higher in depression than those who did not, with this disparity gradually widening over time. This result implies that the psychological pressures stemming from childbirth might intensify with the passage of time. A deeper investigation into the root causes of this issue, coupled with clearer supportive strategies, can help parents better navigate the psychological challenges brought by childbirth and enhance their willingness to give birth.

### Model specification

To study the reverse causality, spillover effects, and lagged effects of first-time childbirth on parents’ mental health, we construct the following fixed-effects model:


$$Y_{ihvt}=\beta_{\text{0}}+\beta_{\text{1}}X_{ihvt}+\beta_{\text{2}}C_{ihvt}+\alpha_{i}+\delta_{hv}+\lambda_{t}+\varepsilon_{ihvt}$$ (1)

Where:i represents the individual,h represents the family,v represents the community,t represents time.$Y_{ihvt}$ is the dependent variable,$X_{ihvt}$ is the independent variable,$C_{ihvt}$ comprises control variables at the individual, family, and village levels, including age, gender, educational level, family financial status, marital satisfaction, and child-rearing expenses.$\alpha_{i}$, $\delta_{hv}$, $\lambda_{t}$ respectively represent the fixed effects at the individual, family (village), and time levels. $\varepsilon_{ihvt}$ denotes the random error term aggregated at the family level, allowing for correlations among individuals within the same family.

Event study is a tool widely used in the social sciences for causal inference and policy evaluation. We employ the event study method to investigate the core issue of the impact of first-time childbirth on parents’ psychological health, examining the parallel trend hypothesis and the dynamic causal effect of first-time childbirth policies.


$$Mentalhealth_{ihvt}\;=\sum_{\tau =-4,\tau =-1}^{\text{4}}\beta_{\tau }\;\times 1(FirstchildPeriod_{hvt\;}=\tau )+\alpha_{i}+\delta_{hv}+\lambda_{t}+\varepsilon_{ihvt}\;$$ (2)

Here, i represents the individual, h represents the family, v represents the community,t represents time, $Mentalhealth_{ihvt}$ stands for the happiness or depressive emotions of parents.τ=−4,−3,−2,0,1,2,3,4, where the interval between every two consecutive periods is two years.Period_-1_ (τ=−L, the baseline period) is used as a reference point in the model, and is therefore omitted. Period_0_ (τ=0) is the period of first childbirth.$FirstchildPeriod_{hvt\;}$ represents the relative period of the family h in year T to the baseline period. L(ExpropriatedPeriodhvt=τ) indicates whether the family h was before or after the baseline period in year T,The error term $\varepsilon_{ihvt}$ is clustered at the family level. Expropriated period represents the year in which the first child was born.

### Endogeneity and instrumental variable selection

First childbirth is a significant event that greatly impacts parents’ psychological health. At the same time, psychological health influences the decision to have a child, suggesting a possible reverse causal relationship between first-time childbirth and parents’ psychological health. Additionally, specific sociocultural contexts and village childbirth trends may directly influence the decision for the first childbirth, leading to omitted variable bias.

To address these endogeneity issues, we use the one-child birth rate in the same village as an instrumental variable for the decision of first-time childbirth. The one-child birth rate in the same village refers to how often families in a particular village have their firstborn in a single year. Here, “village” refers to a smaller community within a town or city.

Our choice is based on empirical results showing that the one-child birth rate in a given village is significantly related to a family’s decision to have their first child. When most families in a village choose to have one child, other families in the same village are more likely to make a similar decision. While the one-child birth rate in the same village is closely related to a family’s decision to have their first child, it does not directly affect the psychological health of the parents in that family. This makes it a suitable instrumental variable.

To validate the effectiveness of this instrumental variable, we conducted a first-stage estimation. The results indicate that the coefficients for the one-child birth rate in the same village are all significant, with F-values in each model far greater than 10. This demonstrates a strong correlation between the instrumental variable and the endogenous explanatory variable. The one-child birth rate in the same village, as used in this study, demonstrates its validity and helps address potential endogeneity concerns, providing a more robust method for estimating the impact of first childbirth on parents’ psychological health.

## Estimation results and analysis

During the model estimation phase, there are two potential issues that might influence the results. Firstly, after controlling for individual and time fixed effects, there is a changing trend over time between first childbirth and the outcomes related to psychological health. Secondly, if individuals in the control group, who haven’t experienced first childbirth, are influenced by their neighbors’ decision to have their first child, the baseline estimates might be biased towards zero. Next, we will discuss several strategies to address these concerns and validate the identified assumptions.

### Examination of non-time-varying and time-varying features

Initially, this paper examines whether there are individual or family factors that influence a family’s decision to have their first child. By conducting a comparative analysis between first-time parent groups’ non-time-varying and time-varying features, we identify which individual or family factors might influence family birth decisions, avoiding confounding factors and reverse causality leading to endogeneity problems.

Table 2 presents the non-time-varying characteristics of individuals and families who have had their first child compared to the control group. Panel A focuses on individual-level characteristics. Panel B focuses on family-level characteristics. Of note, our analysis compares families who had their first child during the survey year with families who did not have their first child during the survey year, not families with their first child to families with no children at all. The results show that the decision to have the first child is influenced by a combination of various factors, including age, eduy, finance, spouse_work, and child_fee. Therefore, in the model estimation, we need to further control them to account for the influence of individual and family characteristics on the results.

**Table 2:** The time-unvarying determinants of first childbirth.

	Firstchild					
	Total		No	Yes	MeanDiff	
Variables	Mean	Std.dev	Mean1	Mean2	Diff.	p-Value
Panel A:Individual-level characteristics						
age	45.0662	17.7698	49.8281	34.4875	15.3405	0.0000
eduy	7.4623	4.8302	6.7309	9.0953	−2.3644	0.0000
hukou	0.3637	0.9307	0.3649	0.3610	0.0039	0.3955
ccp	0.1572	0.3640	0.1566	0.1587	−0.0022	0.3441
Panel B:Household-level characteristics						
finance	0.0656	0.2476	0.0631	0.0708	−0.0077	0.0000
marry_co ~ t	0.8639	0.3429	0.8606	0.8661	−0.0055	0.0418
spouse_w ~ k	0.7551	0.4300	0.7938	0.6977	0.0961	0.0000
child_fee	0.4189	0.4934	0.3960	0.4675	−0.0715	0.0000

Note: age = age of each parent; eduy = number of years of education of each parent; hokou = household registration status (rural/urban); ccp = Communist Party membership status of each parent (no/yes);finance = household financial status; marry_co ~ t = marital satisfaction ratings from each parent; spouse_w ~ k = household chore satisfaction ratings from each parent; child_fee = household childcare expenses.

To delve deeper into how time-varying family factors influence the decision to have a first child, this paper employs panel data of families for empirical testing. Table 3 and Table 4 present the regression relationships between first childbirth and various time-varying family factors. As shown in Table 3, the decision to have the first child is largely positively influenced by the fixed assets owned by the family, such as property. In contrast, other economic factors like net family assets, financing constraints, and financial holding situations have limited influence on the childbirth decision.

**Table 3:** The time-varying determinants of first childbirth.

Firstchild (Household-year panel)						
	(1)	(2)	(3)	(4)	(5)	(6)
Net asset (t-1)	0.0229					0.0332
	(−0.0375)					(−0.0446)
Own house (t-1)		0.0650***				0.0726*
		(−0.0239)				(−0.0429)
Constraint (t-1)			−0.0116			0.01
			(−0.0122)			(−0.0182)
Finance hold (t-1)				0.0323		0.0000
				(−0.037)		(−0.0489)
Coresidence (t-1)					0.0603	0.0209
					(−0.0261)	(−0.0512)
Villages	2520	2807	2807	2740	2856	2424
Observations	13229	16745	16745	14045	16968	10647
Adj. R-Squared	0.6326	0.63	0.6296	0.6624	0.6302	0.6953
Individual fixed	--	--	--	--	--	--
Household fixed	Yes	Yes	Yes	Yes	Yes	Yes
Time fixed	Yes	Yes	Yes	Yes	Yes	Yes

Note: *** and * refer to statistical significance at the 1% and 10% levels, respectively; then values in parentheses of static estimation refer to t-statistics. Table 3 presents the effects of various household-level variables on the decision to have a first child using multiple regression models, where each model analyzes the impact of individual variables independently (Models 1–5) and collectively (Model 6), reporting unstandardized regression weights. The term ‘(t-1) ’indicates that variables are measured in the year prior to the birth of the first child. Definitions: ‘Net asset (t-1)’ refers to the family’s total assets (e.g., real estate, vehicles) minus total liabilities (e.g., mortgage loans, car loans) in the year before the first child was born. ‘Own house (t-1)’ refers to whether the family owned any property (e.g., real estate such as houses or land) in the year before the first child was born. ‘Constraint (t-1)’ refers to the family’s financial burdens or challenges (e.g., credit card debts, personal loans in the year before the first child was born. ‘Financial hold (t-1)’ refers to the value of more liquid financial assets such as bank deposits, stocks, and bonds held in the year before the first child was born, emphasizing assets readily convertible into cash. ‘Coresidence (t-1)’ refers to whether one or both grandparents(i.e., parents of the survey respondents) reside in the same household loans in the year before the first child was born.

**Table 4:** The time-varying determinants of first childbirth.

	Firstchild (Individual-year panel)							
	(7)	(8)	(10)	(11)	(12)	(13)	(14)	(15)
happiness (t-1)	−0.0019							−0.0059
	(−0.0192)							(−0.042)
Depress (t-1)		−0.0071***						−0.0243
		(−0.0025)						(−0.0195)
live content (t-1)			−0.0134					−0.0045
			(−0.0092)					(−0.0567)
Health (t-1)				−0.0012				−0.0018
				(−0.0073)				(−0.0409)
Marry content (t-1)					−0.0122			0.0063
					(−0.0453)			(−0.0512)
Spouse economy (t-1)						−0.0437		−0.0389
						(−0.0381)		(−0.0424)
Spouse housework(t-1)							−0.0247	−0.0117
							(−0.0352)	(−0.0371)
Villages	3182	4362	4162	4517	2272	2272	2272	2265
Observations	35550	61121	60147	63999	16225	16219	16220	16158
Adj. R-Squared	0.4737	0.6469	0.6462	0.6517	0.5908	0.5912	0.5909	0.5935
Individual fixed	Yes	Yes	Yes	Yes	Yes	Yes	Yes	Yes
Household fixed	--	--	--	--	--	--	--	--
Time fixed	Yes	Yes	Yes	Yes	Yes	Yes	Yes	Yes

Note: *** refers to statistical significance at the 1% level; the values in parentheses of static estimation refer to t-statistics. Table 4 explores the influence of individual-level variables on the decision to have a first child through separate regression models for each variable (Models 1−4) and a comprehensive model integrating all variables (Model 5), with results shown as unstandardized regression weights. The ‘(t-1)’ notation signifies that all measurements are from the year before the first child’s birth. Definitions: ‘Happiness (t-1)’ refers to the happiness ratings from each parent the year before they had their first child. ‘Depression (t-1)’ refers to the depression ratings from each parent the year before they had their first child. ‘Live content (t-1)’ refers to the life satisfaction ratings from each parent the year before they had their first child. ‘Health (t-1)’ refers to the health status of each parent the year before they had their first child. ‘Marry content (t-1)’ refers to the marital satisfaction ratings from each parent the year before they had their first child. ‘Spouse economy (t-1)’ refers to the economic contribution of each parent. ‘Spouse housework (t-1)’ refers to the housework contribution of each parent

Further, we investigated how individual time-varying factors influence the decision to have a first child. The result shows that families who experienced depressive symptoms in the previous year might be more cautious about deciding to have their first child. For other individual variables from the previous period, such as life satisfaction, health status, marital satisfaction, spouse’s economic and housework contribution, etc., their relationships with the decision to have the first child are mostly insignificant in most models.

Overall, the decision to have the first child is significantly influenced by the family’s depressive symptoms from the previous period. In contrast, other emotional and mental health factors have limited influence on the childbirth decision. This indicates that there’s no reverse causality between individual time-varying factors and the decision to have the first child, providing data support for subsequent research.

### Testing for spillover effects

If the mental health of individuals who haven’t experienced first childbirth is influenced by their neighbors having their first child, the baseline estimates would be biased towards zero. To avoid this bias, we will discuss and verify whether there are spillover effects between first childbirth and parental mental health.

The results from Table 5 indicate that first childbirth in other families within the same village does not produce significant spillover effects on the mental health of parents in the subject family. Even after introducing the variable of the subject family’s first childbirth, the spillover effect remains insignificant. At the same time point, there’s no significant difference in mental health changes between the treatment and control groups within the same village. This suggests that while first childbirth directly affects the mental health of mothers, this impact does not significantly spill over into the broader community or region, providing a basis for policy formulation and mental health interventions.

**Table 5:** Spillover effects of first childbirth on mental health.

	Happiness	Depression	Happiness	Depression
	(1)	(2)	(3)	(4)
Firstchild	−0.0565	0.0536***		
	(0.0468)	(0.0161)		
Ratio of treated villagers	−0.1450	0.0317	−0.1515	0.0366
	(0.0971)	(0.0311)	(0.0968)	(0.0311)
Villages	16784	18041	16784	18041
Observations	93194	147191	93194	147191
Adj. R-Squared	0.3670	0.3165	0.3670	0.3164
Individual fixed	Yes	Yes	Yes	Yes
Time fixed effect	Yes	Yes	Yes	Yes

Notes:*** refers to statistical significance at the 1% level; the values in parentheses of static estimation refer to t-statistics.Table 5 examines the potential spillover effects of first childbirth on parental mental health within villages. Columns (3) and (4) estimate the proportion of treated residents within the village, focusing on families who had their first child. The ‘Ratio of treated villagers’ measures the proportion of such families in the village. The comparison between treatment and control groups within the same village helps control for village-specific factors affecting mental health. Column (1): Impact of First Childbirth on Happiness. Column (2): Impact of First Childbirth on Depression. Column (3): Ratio of Treated Villagers on Happiness.Column (4): Ratio of Treated Villagers on Depression.

### Baseline estimation results

To further analyze the relationship between first childbirth and parents’ mental health, this paper conducts empirical analysis based on formula 0.1, with results presented in Table 6.

**Table 6:** Impact of First Childbirth on Parental Happiness and Depressive Mood.

	Happiness							
	(1)	(2)	(3)	(4)	(5)	(6)	(7)	(8)
Firstchild	−0.0444	−0.1330*	−0.0471	−0.0080	−0.0419	−0.1024**	−0.0970**	−0.0217
	(0.0333)	(0.0687)	(0.0407)	(0.0341)	(0.0579)	(0.0460)	(0.0458)	(0.0485)
Villages	45610	45610	45610	44261	39638	37802	22788	22925
Observations	91207	91207	91207	88610	62806	57610	45227	45980
Adj. R-Squared	0.3813	−0.8473	0.3656	0.3898	0.3834	0.4365	0.3550	0.4083
	**Depression**							
	(1)	(2)	(3)	(4)	(5)	(6)	(7)	(8)
Firstchild	0.0544***	0.0044	0.0259	0.0379**	0.0036	0.0860***	0.0762***	0.0412**
	(0.0161)	(0.0315)	(0.0180)	(0.0160)	(0.0380)	(0.0205)	(0.0221)	(0.0207)
Villages	18041	18041	18041	17548	16218	15099	15997	16298
Observations	147191	147191	147191	136506	62860	65884	70152	77024
Adj. R-Squared	0.3165	0.1586	0.3738	0.2883	0.3783	0.4628	0.3151	0.3109
Individual fixed effects	Yes	Yes	Yes	Yes	Yes	Yes	Yes	Yes
Time Fixed effect	Yes	No	No	No	Yes	Yes	Yes	Yes
Family FEs*time FEs	No	Yes	No	No	No	No	No	No
Village FEs*time FEs	No	No	Yes	No	No	No	No	No
Time varying control	No	No	No	Yes	No	No	No	No
time invariant control	No	No	No	No	Yes	No	No	No

Notes:*** refers to statistical significance at the 1% level; the values in parentheses of static estimation refer to t-statistics. Table 6 presents the regression models analyzing the impact of first childbirth on parental happiness and depressive mood. Columns (1) through (8) contain unstandardized regression weights (b), indicating the absolute effect of each variable on the dependent variable, holding all other variables constant. The values in parentheses are the t-statistics, which measure the statistical significance of the regression coefficients. (1) = Baseline model; (2) = Including family and time interaction terms; (3) = Including village and year interaction terms; (4) = Including time-varying control variables; (5) = Including time-invariant control variables with time interaction terms; (6) = Considering only the last 2 years; (7) = Considering only a random sample of mothers; (8) = Considering only a random sample of fathers.

Impact on parental happiness. As shown in the upper part of Table 6, first childbirth might have a negative impact on parental happiness. In column (1), the regression coefficient between “Firstchild” and parental happiness is −0.0444, yet it lacks statistical significance. When family and time interaction terms are included in column (2), this coefficient increases to −0.1330 and becomes significant at the 10% level. This suggests that first childbirth may reduce family members’ happiness. To verify the robustness of this relationship, we employed several strategies. In columns (3) to (5), we introduced village and year interaction terms, time-varying control variables, and time-invariant variables with time interaction terms, and these model results further support the baseline regression outcomes. Columns (6) to (8) further test robustness, retaining samples from the most recent two periods, samples of mothers, and samples of fathers. These estimated coefficients are consistent with baseline results, confirming the robustness of the baseline regression.

Impact on parental depressive mood. As can be seen from the lower part of Table 6, first childbirth has a positive correlation with parents’ depressive mood. Column (1) demonstrates a positive relationship between first childbirth and family members’ depressive mood, significant at the 1% level. When only family and time fixed effects are introduced in column (2), the significance of the relationship between first childbirth and depressive mood weakens, but the overall trend remains positive. In columns (3) to (8), we employed various robustness testing methods, including introducing village and year interaction terms, time-varying control variables, time-invariant variables with time interaction terms, retaining the most recent two-period samples, and samples of mothers and fathers. The empirical outcomes from these methods align with the baseline results, confirming their robustness. In conclusion, first childbirth might lead to a decrease in parental happiness and an increase in depressive mood. These findings provide crucial evidence for further studying the potential impact of childbirth decisions on family members’ mental health.

### Placebo test

To further verify the impact of first childbirth on parental happiness and depressive mood, and to ensure that these results are not caused by other unobservable factors, this paper further analyzes the randomness of the impact of first childbirth. Based on the distribution of the “Firstchild” variable in the baseline regression, a placebo test was conducted. By randomly sampling to construct 1,000 “pseudo-childbirth dummy variables” and re-estimating based on the previous model, we paid special attention to the distribution of their coefficients and P-values. The left chart “Happiness” and the right chart “Depression” in Fig 3 respectively display the estimated coefficients and P-values distribution of the “pseudo-childbirth dummy variable” for happiness and depressive mood.

In Fig 3, the average of the estimated coefficients is close to 0, and the distribution of most estimated coefficients aligns closely with a normal distribution. Most of these coefficients are not significant, with P-values mainly distributed above 0.10, meaning they are not significant at the 10% level. This suggests that the impact of first childbirth on the happiness and depressive mood of family members is not coincidental. Through the placebo test, we further confirmed the robustness of the effects of first childbirth on the happiness and depressive mood of family members, ensuring that the empirical results are not caused by other unobservable factors.

**Fig 2:**
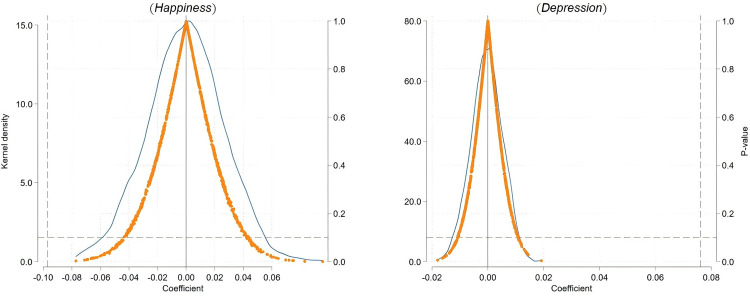
Distribution of placebo test. Note: Fig 2 displays the results of the placebo test conducted to verify the robustness of the observed effects of first childbirth on parental happiness and depressive mood. The blue line represents the distribution of estimated coefficients for happiness using 1,000 pseudo-childbirth dummy variables, while the orange line represents the distribution of estimated coefficients for depressive mood. The placebo test ensures that the observed effects are not driven by random chance or unobservable factors, confirming the robustness of our findings.

### Estimation of dynamic effects

**Fig 3:**
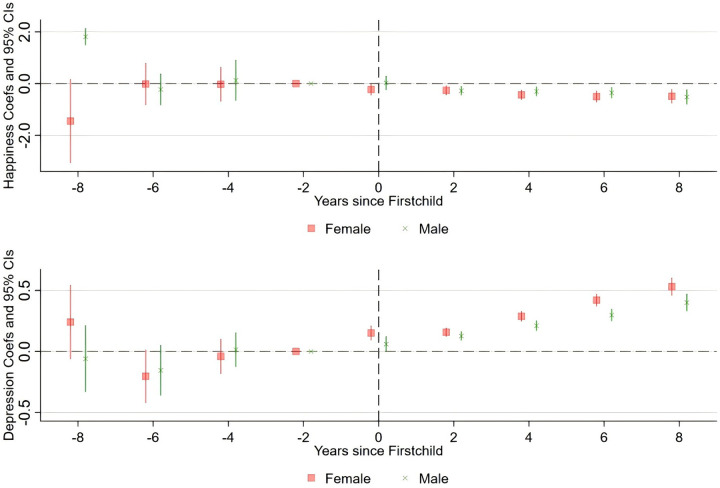
Dynamic effects of first childbirth on mental health.

To systematically study the impact of first childbirth on parental mental health, we used Model 0.2 to estimate its dynamic effects and plotted the estimated coefficients with their respective 95% confidence intervals in Fig 3. Given the varying impacts of first childbirth on parents’ mental health, to address the potential biases caused by heterogeneity, this paper reports the effects separately for fathers and mothers. Fig 3 shows the trends in happiness and depressive mood of parents before and after childbirth. The red line represents mothers, and the green line indicates fathers. The result shows that first childbirth has a significant impact on mothers’ happiness and depressive mood, while the influence on fathers is relatively minor.

### Endogeneity test

As shown in Table 7, the impact of first childbirth varies for fathers and mothers in terms of happiness and depressive mood. Each column respectively applies the fixed effects model and the instrumental variable method for estimation. Columns (1), (3), (5), and (7) present results based on the fixed effects model, while columns (2), (4), (6), and (8) show results from the instrumental variable method, aiming to reveal the causal effect of first childbirth on parental mental health. This study uses the one-child birth rate in the same village as an instrument. The results of the first stage further confirm the validity of this instrument. Additionally, the F-value in each model is much greater than 10, indicating a strong correlation between the instrument and the endogenous explanatory variable. In all models, we control for time and individual fixed effects to eliminate unobserved time and individual heterogeneity.

**Table 7:** Impact of First Childbirth on Parental Mental Health: An Instrumental Variable Approach.

	Happiness				Depression			
	Female		Male		Female		Male	
	OLS	2SLS	OLS	2SLS	OLS	2SLS	OLS	2SLS
Firstchild	−0.0881*	−0.2257***	−0.0126	0.0803	0.0178	0.0736***	0.0079	0.1408***
	(0.0454)	(0.0781)	(0.0469)	(0.0809)	(0.0121)	(0.0239)	(0.0129)	(0.0254)
Control	Yes	Yes	Yes	Yes	Yes	Yes	Yes	Yes
IV estimation: First stage								
Treated_ratio		0.7594***		0.7770***		0.7616***		0.7569***
		(0.0109)		(0.0121)		(0.0102)		(0.0106)
F-value		4888.33		4154.57		5542.62		5093.59
Observations	42943	42943	45408	45408	75204	59223	76568	65518
Time Fixed effect	Yes	Yes	Yes	Yes	Yes	Yes	Yes	Yes
Individual fixed	Yes	Yes	Yes	Yes	Yes	Yes	Yes	Yes

Notes: *** refers to statistical significance at the 1% level; the values in parentheses of static estimation refer to t-statistics.

By comparing the results of the fixed effects model and the instrumental variable method, we find that the estimates from the instrumental variable method are often larger and more statistically significant. This might be because the instrumental variable method more effectively corrects potential endogeneity issues. First childbirth has a significant positive effect on the depressive mood of parents. For mothers, first childbirth has a significant negative impact on happiness, while the impact on fathers’ happiness is not significant in both model estimates. The results suggest that first childbirth has a significant effect on the depressive mood of both parents and reduces happiness significantly for mothers, but not for fathers. These findings have important implications for policy-making and practice, highlighting the need for greater attention and care for the mental health of new parents, especially mothers.

## Discussion

Against the backdrop of the demographic transition theory, as nations shift from traditional high birth and death rates to modern low birth and death rates, there have been significant changes in the structure and function of families. During this transition, the event of first-time childbirth, as a crucial milestone in the family lifecycle, has drawn widespread attention for its impact on the psychological well-being of parents. We will explore the impact of first-time childbirth on parents’ mental health from two aspects. On the one hand, there is heterogeneity in the impact of first-time childbirth on the mental health of fathers and mothers. On the other hand, there is heterogeneity in the impact of first-time childbirth on parents’ mental health across different social contexts.

Firstly, differences between fathers and mothers: Throughout the demographic transition, while both fathers and mothers feel the impacts of first-time childbirth, mothers, due to physiological, cultural, and societal differences, often endure greater stress [46,47]. Heterogeneity in effects: The impact of first-time childbirth on parents reveals pronounced heterogeneity and spillover effects. Younger parents might find it easier to adapt to the arrival of a newborn, whereas middle-aged and older parents could experience heightened psychological stress due to more pronounced economic and health challenges [48]. Urban parents, generally equipped with better economic and educational resources, might also grapple with intensified work and life pressures [49]. In contrast, rural parents might face significant economic challenges but could benefit from greater support from their community and extended family. Highly-educated parents might have easier access to child-rearing information and resources but might also have loftier parenting expectations [50]. In contrast, parents with lower educational attainment might face challenges in accessing child-rearing knowledge and skills but might find more robust support within their families and communities [51]. In certain regions, owing to traditional gender preferences (“preference for boys over girls”), the birth of a girl might impose added psychological stress on parents, affecting their happiness and mood [52]. While both fathers and mothers are influenced by first-time childbirth, due to physiological, cultural, and societal role differences, mothers often bear a heavier burden, especially when the child is a girl [53].This data validates that rural residents experience much larger fluctuations in mental health when facing childbirth transitions compared to urban residents. This might be attributed to economic conditions, social support systems, and cultural expectations in rural areas [54,55].Against the backdrop of a decelerating economic growth in rural areas, there’s a reduced demand for labor, especially affecting migrant workers. Therefore, rural women might face intensified life pressures after childbirth, not solely due to physical and psychological changes from childbirth, but also potential loss of a stable income source. In cities, although decelerating economic growth also impacts residents’ mental health, urban residents, benefiting from more societal resources and support, experience relatively lesser psychological pressure post-childbirth [56].To better understand and support the mental well-being of couples post-childbirth, there’s a need to delve into their economic and societal backgrounds. Particularly in the current scenario of decelerating economic growth, the challenges and needs concerning the mental health of urban and rural residents differ [57]. The impact of the first childbirth on the mental health of urban and rural residents varies significantly. Especially in rural areas, women often face intensified pressures and challenges, necessitating more support and resources from the government and society [58]. Concurrently, urban men also face a series of challenges, especially in economic and career aspects, resonating with the “optimism paradox” concept by Gilbert et al. (2021) [59].

In summary, the demographic transition has not only profoundly influenced the structure and economy of nations and societies but has also ushered in significant changes in the lifestyles, values, and mental health of families and individuals. Approaching from the perspective of first-time childbirth, this study offers deep insights into how this transformation affects the psychological well-being of parents.

## Conclusions

First-time childbirth has profound effects on the psychological health of parents, influenced by various socio-economic and cultural factors. Our findings reveal that while the birth of a child brings joy and fulfillment, it also introduces significant psychological pressures. Mothers tend to experience a notable decline in happiness and an increase in depressive symptoms post-childbirth, largely due to the physical, emotional, and social adjustments required. Fathers also exhibit a significant increase in depressive symptoms, though their happiness levels remain statistically unchanged, a pattern that may be shaped by societal roles and expectations.

The study highlights the necessity for targeted support and intervention strategies to assist parents in navigating the challenges associated with first-time childbirth. Providing adequate mental health resources and fostering a supportive environment for both parents can mitigate the adverse effects on their well-being.

Understanding the distinct impacts on mothers and fathers is crucial for developing comprehensive policies that address the specific needs of each parent. Future research should continue exploring these dynamics to further inform policy and support measures, ultimately promoting the mental health and well-being of new parents.

## Supporting information

### Mechanism ransmission analysis

The baseline regression results indicate that first childbirth has a significant impact on parental mental health. To delve deeper into its potential transmission mechanisms, we conducted phased empirical tests based on literature and real-world observations. This paper identifies the following four key transmission mechanism variables: chronic diseases, work income, job opportunities, and confidence in the future. We examined how first childbirth affects each mechanism variable, followed by a regression analysis on the relationships between these variables and parental mental health.

#### Chronic diseases.

The relationship between chronic diseases and mental health has been well-documented in previous studies. Engel (1977) [45] proposed that chronic diseases significantly affect mental health, leading to various psychological issues. On the one hand, patients with chronic diseases experience reduced work efficiency due to frequent medical visits or lack of energy [60], and are forced to choose low-intensity and low-income jobs [61]. On the other hand, the direct medical costs incurred by chronic diseases further squeeze the space for income accumulation [62], triggering dual anxiety about health and the economy and falling into a vicious cycle of lacking a sense of control over the future.This severely impacts mental health, giving rise to various psychological issues. According to data from Table 8, after the first childbirth, mothers face a significant increase in the risk of chronic diseases. In contrast, the risk growth for fathers is not evident. Table 9 further reveals the correlation between chronic diseases and feelings of happiness and depression: chronic diseases are significantly negatively correlated with happiness and positively correlated with depressive mood.

**Table 8:** Impact of Bearing the first Child on Mechanistic Variables.

	Chronic		Work income		Work opportunity		Confident	
	Female	Male	Female	Male	Female	Male	Female	Male
Firstchild	0.0157***	−0.0012	−0.0709**	0.0500*	−2.4426***	−2.8265***	−0.0265*	−0.0057
	(0.0049)	(0.0049)	(0.0295)	(0.0285)	(0.8635)	(0.8312)	(0.0155)	(0.0169)
Control	Yes	Yes	Yes	Yes	Yes	Yes	Yes	Yes
Observations	75215	76584	31919	36120	35487	41865	74995	76490
Adj. R-Squared	0.2508	0.2468	0.2977	0.3352	0.3881	0.3684	0.2855	0.3394
Time Fixed effect	Yes	Yes	Yes	Yes	Yes	Yes	Yes	Yes
Individual fixed	Yes	Yes	Yes	Yes	Yes	Yes	Yes	Yes

Notes: *** refers to statistical significance at the 1% level; the values in parentheses of static estimation refer to t-statistics.Table 8 presents the impact of first childbirth on various mechanism variables using unstandardized regression weights (b). Columns represent the following: (1) Chronic = Likelihood of chronic diseases; (2) Income = Work income; (3) Job Opportunities = Opportunities for employment; (4) Confidence = Level of confidence in the future. Individual and time fixed effects are included.

**Table 9:** Impact of Mechanistic Variables on Parental Psychological Well-being.

	Happiness				Depression			
	(1)	(2)	(3)	(4)	(5)	(6)	(7)	(8)
Chronic	−0.0733***				0.1290***			
	(0.0275)				(0.0075)			
Work income		0.1399***				−0.0328***		
		(0.0173)				(0.0033)		
Work opportunity			0.0008*				−0.0005***	
			(0.0004)				(0.0001)	
Confident				0.4781***				−0.1124***
				(0.0123)				(0.0029)
Control	Yes	Yes	Yes	Yes	Yes	Yes	Yes	Yes
Observations	88370	41949	50340	88345	151799	68039	77352	151485
Adj. R-Squared	0.3659	0.4350	0.4312	0.3971	0.3926	0.4200	0.4105	0.4022
Time Fixed effect	Yes	Yes	Yes	Yes	Yes	Yes	Yes	Yes
Individual fixed	Yes	Yes	Yes	Yes	Yes	Yes	Yes	Yes

Notes: *** refers to statistical significance at the 1% level; the values in parentheses of static estimation refer to t-statistics. Table 9 presents the impact of various mechanism variables on parental mental health using unstandardized regression weights (b). Columns represent the following: (1) Happiness = Level of happiness; (2) Depressive Mood = Level of depressive mood. Individual and time fixed effects are included.

The childbirth process might lead women to undergo a series of physiological changes, elevating their risk for certain chronic diseases. For instance, Evenson et al. (2009) [63] highlighted that pregnancy and childbirth could induce changes in the endocrine, immune, and metabolic systems, potentially correlating with a rise in the incidence of chronic diseases. Furthermore, the physical and psychological stress after childbirth, combined with the daily care of a newborn, might further impact a woman’s health [13]. This aligns with Engel’s model. Consequently, women might face heightened health risks after childbirth, further adversely affecting their mental health.

#### Work income.

Income, as a crucial indicator to measure the economic status of individuals or families, has always been a hot topic in social science research in relation to mental health. Festinger (1954) [64] proposed the “Social Comparison Theory,” emphasizing that individuals often assess their social status by comparing their economic conditions with others. Cheung & Lucas (2016) [65] further confirmed that when an individual’s work income is below their social circle or expected standards, their mental health is likely to be negatively affected. Specifically, a substantial disparity between personal income and the societal average may trigger a sense of relative deprivation. Even among individuals in good health, anxieties about “social stratification rigidity” can undermine their confidence in the future.

Data from Table 8 indicates that after the first childbirth, women’s work income significantly decreases, while men’s slightly increases. Table 9 further reveals the correlation between work income and individual mental health, especially happiness and depressive mood. Notably, work income has a significant positive correlation with happiness and a significant negative correlation with depressive mood. The significant decline in mothers’ work income after childbirth could be attributed to traditional cultural and social structures, where mothers are often expected or compelled to stay at home to care for their children, subsequently limiting their career opportunities and working hours [66]. Prolonged family caregiving responsibilities might make it challenging for women to maintain full-time jobs or to secure promotions and salary increases in the workplace [67]. Furthermore, a decrease in family income, especially in dual-income households, could lead to increased financial stress. This economic pressure translates into a psychological burden, impacting the mental health of family members [68].

#### Job opportunities.

In the context of today’s diverse society, the roles individuals bear are increasingly complex. Consequently, how to appropriately allocate time and energy among these roles has gradually become a critical factor affecting mental health. Goode (1960) [69] deeply explored this topic in his “Role Strain Theory,” emphasizing that individuals, when faced with the dilemma of choosing and balancing among multiple roles, are prone to experiencing psychological stress and conflict. For new parents, the potential conflict between work responsibilities and family caregiving duties might limit their job opportunities—a perspective supported by research from Nomaguchi & Milkie (2020) [8]. This psychological strain stemming from role conflicts might adversely affect their overall mental well-being.

Specific data, as shown in Table 8, indicates that new parents, regardless of gender, face a significant reduction in job opportunities after their first childbirth. Further, from Table 9, it’s evident that this decrease in job opportunities correlates positively with individual happiness and negatively with depressive moods.

The underlying reason might be the immense caregiving pressure on parents, especially mothers, during the early stages of a child’s life. This pressure could compel them to make trade-offs between their careers and families. Particularly during pivotal career advancement phases, they might miss out on some significant job or promotion opportunities. Not only might this impact their financial income, but more critically, it could profoundly affect their job satisfaction, self-worth, and mental health. Such role conflicts and choices undoubtedly intensify their psychological and social adaptation pressures, possibly further deteriorating their mental health.

#### Confidence in the future.

Bandura’s (1977) [70] theory of self-efficacy suggests that an individual’s beliefs about their capabilities influence their emotions, thoughts, and actions. Individuals with strong confidence in the future are more inclined to engage in health-promoting behaviors, such as regular exercise and a balanced diet [71], and demonstrate higher risk tolerance in career development, proactively seeking challenging work opportunities to achieve higher income returns, thereby promoting the development of individual mental health. Data from Table 8 indicates that, after the first childbirth, women’s confidence in the future slightly declines, though the decline is not significant. In contrast, men’s confidence in the future remains largely unchanged. Further, Table 9 reveals the relationship between confidence in the future and mental health. A notable positive correlation suggests that individuals with optimistic expectations about the future significantly experience elevated happiness, while their depressive moods markedly decrease.

Taking everything into account, the first childbirth, as a pivotal event in life, brings a series of physiological, psychological, and social challenges to women. For first-time mothers, the challenges and uncertainties during this period might impact their confidence in the future. Factors such as childcare responsibilities, interruptions in career progression, and changes in the family’s financial situation could potentially affect women’s mental health. From the perspectives of Bandura’s self-efficacy theory, this waning confidence in the future, especially when confronted with new childbirth challenges, could further intensify women’s psychological stress, detrimentally affecting their mental health.

The research findings reveal that after the first childbirth, the risk of mothers contracting chronic diseases significantly increases, negatively impacting their mental health. After giving birth, mothers’ work incomes notably decrease, correlating negatively with their mental health. Childbirth affects job opportunities for parents; reduced job opportunities could have adverse effects on their mental well-being. After the first childbirth, women’s confidence in the future slightly declines, which might further influence their mental health. Overall, the first childbirth has a more pronounced impact on mothers’ mental health. These effects might be associated with biological changes, societal expectations, and economic pressures. To enhance parents’ mental health, further research into these transmission mechanisms is necessary, along with the implementation of appropriate measures.

## Extended analysis

### Age

Further in this paper, we analyze the impact of the first childbirth on the mental health of parents from different age groups. Referring to the World Health Organization’s age categorization, couples 44 years old and below are defined as “young couples”, while those aged between 45 and 59 are termed “middle-aged couples”. As shown in Table 10, among young couples, women’s happiness significantly decreases after their first childbirth, while the decrease for men is less pronounced. Conversely, among middle-aged couples, women’s happiness after their first childbirth is positively correlated, while the change for men is almost negligible. Regarding depressive moods, young women show a significant increase after their first childbirth. In contrast, among middle-aged couples, women’s depressive moods decrease, and the reduction for men is even more pronounced. These differences may relate to the physical and psychological pressures after childbirth, balancing work and family, and societal expectations for male and female roles.

**Table 10:** The Impact of First-time Childbirth on Parents’ Mental Health: age Heterogeneity.

	Happiness				Depression			
	Young couple		Middle-aged couple		Young couple		Middle-aged couple	
	Female	Male	Female	Male	Female	Male	Female	Male
Firstchild	−0.1704***	−0.0779	0.1393	−0.0106	0.0906***	0.0545***	−0.0995*	−0.1533***
	(0.0491)	(0.0546)	(0.1701)	(0.1156)	(0.0153)	(0.0168)	(0.0534)	(0.0435)
Control	Yes	Yes	Yes	Yes	Yes	Yes	Yes	Yes
Observations	22251	24642	23853	24284	41439	44551	46359	45253
Adj. R-Squared	0.3966	0.4028	0.3477	0.3863	0.3046	0.3128	0.3022	0.2984
Individual fixed	Yes	Yes	Yes	Yes	Yes	Yes	Yes	Yes
Time fixed	Yes	Yes	Yes	Yes	Yes	Yes	Yes	Yes

Notes: *** refers to statistical significance at the 1% level; the values in parentheses of static estimation refer to t-statistics.Table 10 presents the impact of first childbirth on various outcomes using unstandardized regression weights (b). Individual and time fixed effects are included.

For young couples, the significant decline in women’s happiness after their first childbirth may be linked to physical changes after childbirth and the pressure to balance family and career. The relatively smaller decrease in men’s happiness might be because they face less pressure in familial roles and societal expectations after childbirth. Conversely, among middle-aged couples, the rise in women’s happiness after their first childbirth might stem from being better prepared and having clearer expectations about childbirth at this age. Additionally, they might have already found a balance between career and family. The negligible change in men’s happiness could be due to life stability at this age and psychological preparedness for childbirth.

In terms of depressive moods, young women exhibit a significant increase after their first childbirth. This might be attributed to the multiple role conflicts they face post-childbirth, physical recovery, and relationship changes with their partners. For middle-aged couples, women’s depressive moods lighten, possibly because they are more psychologically adjusted to childbirth at this age and receive more familial support. The significant decrease in men’s depressive moods might be attributed to increased job and family satisfaction during middle age, coupled with a positive attitude towards childbirth.

Childbirth is not just a physiological process; it’s a complex experience involving physical, psychological, and social factors. As emphasized by Bilgiç et al. (2021) [72], psychological changes during childbirth are particularly evident in urban China. With the experience of childbirth, couples might face role transitions and pressures, especially in young families. Schofield et al. (2013) [73] pointed out in their study that, especially in the post-one-child-policy era, young parents often face role conflicts between family and work. Moreover, for middle-aged families, Gao et al. (2022) [74] found that childbirth has a positive impact on women’s career aspirations, aligning with our findings. This might be because women at this age stage are better prepared for childbirth and might have found a balance between career and family. On the psychological front, Lebano et al. (2020) [75] explored why modern parents feel stressed yet hold optimistic views, termed the “optimism paradox”. Their research offers insights into the psychological changes in parents post-childbirth. Additionally, Eriksen (2022) [76] emphasized the close relationship between hormonal changes post-childbirth, societal pressures, and mental health.

In summary, to support couples’ mental health post-childbirth, we need to consider their life stage, physiological and psychological preparedness, and socio-cultural background. Especially for young couples, societal and familial support is crucial, assisting them in addressing the multifaceted challenges post-childbirth and achieving harmonious family and career development.

### Urban vs. Rural

The economic and social development disparities between urban and rural areas in China remain significant, especially in terms of resident income and living conditions, reflecting evident differentiation. Due to the long-term impacts of the dual economic structure and the household registration system, the issues between urban and rural areas are increasingly pronounced. Against this backdrop, this study further categorizes individuals based on their household registration to delve deeply into the differential impacts of the first childbirth on the mental health of rural and urban residents. Estimation results are shown in Table 11, comparing the effects of first childbirth on the mental health of men and women in urban and rural areas. For this analysis, we consider “rural” and “urban” as two primary residential environment categories.

**Table 11:** The Impact of First-time Childbirth on Parents’ Mental Health: Urban-Rural Heterogeneity.

	Happiness				Depression			
	Rural		Urban		Rural		Urban	
	Female	Male	Female	Male	Female	Male	Female	Male
Firstchild	−0.1343**	−0.0692	−0.0772	−0.0838	0.0562***	0.0108	0.0069	0.0828***
	(0.0591)	(0.0624)	(0.0901)	(0.0901)	(0.0154)	(0.0173)	(0.0265)	(0.0246)
Control	Yes	Yes	Yes	Yes	Yes	Yes	Yes	Yes
Observations	18103	20274	5568	5901	33254	35562	10592	10647
Adj. R-Squared	0.3782	0.3803	0.4255	0.4180	0.3400	0.3640	0.3550	0.4624
Individual fixed	Yes	Yes	Yes	Yes	Yes	Yes	Yes	Yes
Time fixed	Yes	Yes	Yes	Yes	Yes	Yes	Yes	Yes

Notes:*** refers to statistical significance at the 1% level; the values in parentheses of static estimation refer to t-statistics. Table 11 presents the impact of first childbirth on health and well-being outcomes using unstandardized regression weights (b). Individual and time fixed effects are included.

In terms of happiness: Rural women’s happiness significantly declined by about 13.43% after their first childbirth. This change might be linked to the multiple pressures rural women face post-childbirth concerning physical health, family, and career. This is consistent with the study by Henderson et al. (2005) [77], which pointed out that rural women often face more role transitions and family responsibilities after childbirth. In urban settings, the happiness change for both men and women post-childbirth is not significant. This might be attributed to the abundance of social resources and support in urban areas, allowing individuals to better balance family and career, as emphasized by Hegewisch & Gornick. (2013) [78].

Regarding depressive moods: Depressive symptoms significantly increase among rural women after their first childbirth. This might relate to physiological changes post-childbirth, familial responsibilities, and strained relationships with partners, aligning with findings by Kabir et al. (2014) [79]. Conversely, depressive symptoms among urban men slightly rise post-childbirth, possibly due to economic pressures and career development challenges, as indicated in the research by Nomaguchi & Milkie (2022) [8].

The significant decline in happiness among rural women post-childbirth is substantially more pronounced than that among urban women. Additionally, the increase in depressive symptoms among urban men post-childbirth is much more significant than that among rural men.

### Education

Childbirth, as a pivotal moment in life, profoundly impacts everyone. However, is this impact conditioned by an individual’s educational background? To answer this question, we conducted a detailed analysis of post-childbirth mental health based on education levels, as detailed in Table 12. We can make several key observations:

**Table 12:** The Impact of First-time Childbirth on Parents’ Mental Health: Educational Heterogeneity.

	Happiness				Depression			
	Low Education		High Education		Low Education		High Education	
	Female	Male	Female	Male	Female	Male	Female	Male
Firstchild	−0.0617	0.0374	−0.1098*	−0.1741***	0.0655***	0.0162	0.0538**	0.0435**
	(0.0689)	(0.0723)	(0.0622)	(0.0637)	(0.0199)	(0.0215)	(0.0214)	(0.0215)
Control	Yes	Yes	Yes	Yes	Yes	Yes	Yes	Yes
Observations	14802	16262	9749	10697	29617	30605	18078	20018
Adj. R-Squared	0.3696	0.3665	0.4228	0.4580	0.2757	0.2893	0.4353	0.4290
Individual fixed	Yes	Yes	Yes	Yes	Yes	Yes	Yes	Yes
Time fixed	Yes	Yes	Yes	Yes	Yes	Yes	Yes	Yes

Notes: *** refers to statistical significance at the 1% level; the values in parentheses of static estimation refer to t-statistics. Table 12 presents the impact of first childbirth on economic and social variables using unstandardized regression weights (b). Individual and time fixed effects are included.

(1) Differences in Happiness in Low-Education Background.Among those with a low education level, after the first childbirth, women’s happiness slightly decreases, while men’s happiness tends to rise. This aligns with our previous analysis, indicating that in certain cultural and societal contexts, men might perceive childbirth as a familial responsibility, hence possibly feeling more contented after childbirth [80]. For women, the potential role conflicts between family and work, physiological pressures, and changes due to childbirth may lead to a slight drop in happiness [81].
(2) Differences in Happiness in High-Education Background.Compared to those with a low education level, both women and men with a high education level experience a significant decline in happiness after the first childbirth. This might be attributed to their higher expectations for life and career. Childbirth can lead to greater role conflicts and pressures, especially between work and family [82].
(3) Differences in Depressive Moods in Low-Education Background.Women with a low education background demonstrate a significant rise in depressive moods after the first childbirth. This is in line with our earlier discussion, suggesting that they might face larger multiple role conflicts, pressures of physical recovery, and shifts in relationships with partners [83].
(4) Differences in Depressive Moods in High-Education Background.In the high-education background, both men and women show an increase in depressive moods after childbirth, but the increase is more pronounced among men. This could be related to the career pressures and societal expectations that men with higher education backgrounds confront post-childbirth [84].

The analysis indicates that the psychological impact of childbirth on individuals with a low educational background (especially women) is more pronounced. This suggests that, especially against the backdrop of pressures from childbirth, groups that are already relatively vulnerable in socio-economic status experience more intense psychological shocks. It underscores the importance of tailoring support mechanisms considering educational backgrounds to ensure mental well-being during such transformative life events.

### Child’s gender

From Table 13, we can discern several key insights:

**Table 13:** The Impact of First-time Childbirth on Parents’ Mental Health: child gender Heterogeneity.

	Happiness				Depression			
	Son		Daughter		Son		Daughter	
	Female	Male	Female	Male	Female	Male	Female	Male
Firstchild	0.0159	−0.0249	−0.2076**	−0.0146	0.0742***	0.0869***	0.1151***	0.0323
	(0.0823)	(0.0870)	(0.0844)	(0.0830)	(0.0267)	(0.0304)	(0.0266)	(0.0279)
Control	Yes	Yes	Yes	Yes	Yes	Yes	Yes	Yes
Observations	8551	9116	9449	9511	16517	16450	18188	17401
Adj. R-Squared	0.4043	0.3919	0.3949	0.3771	0.3427	0.3626	0.3454	0.3366
Individual fixed	Yes	Yes	Yes	Yes	Yes	Yes	Yes	Yes
Time fixed	Yes	Yes	Yes	Yes	Yes	Yes	Yes	Yes

Notes: *** refers to statistical significance at the 1% level; the values in parentheses of static estimation refer to t-statistics. Table 13 presents the impact of first childbirth on psychological variables using unstandardized regression weights (b). Standard errors are in parentheses. Individual and time fixed effects are included.

(1) Child’s Gender and Mother’s Happiness.Association between a mother’s happiness and the gender of the child: If the newborn is a boy, the data shows a slight increase in the mother’s happiness, though this change is not statistically significant (coefficient = 0.0159, standard error = 0.0823). This could be related to the traditional family notion of preferring sons over daughters, where sons are often seen as the inheritors of the family lineage. However, accompanying this could be economic responsibilities for the offspring, such as purchasing property or covering wedding expenses, which might influence the mother’s sense of well-being [85]. If the newborn is a girl, the data indicates a significant decline in the mother’s happiness (coefficient = −0.2076, standard error = 0.0844). This might be tied to societal expectations and role perceptions of daughters. In some cultural contexts, daughters might require more care and support, thereby adding more pressure on mothers [86].
(2) Association between a father’s happiness and the gender of the child. Regardless of whether the child is a boy or a girl, there is no significant change in the father’s happiness. This might be tied to traditional roles of men in the family, suggesting that their emotional experience isn’t notably influenced by the gender of the child [87].

(3) Association between a mother’s depressive moods and the child’s gender. Mothers show a significant increase in depressive moods irrespective of the child’s gender. Especially when the child is a girl, her depressive mood significantly rises (coefficient = 0.1151, standard error = 0.0266). This could be associated with post-birth physiological and psychological recovery, changes in familial roles, and expectations and responsibilities towards daughters [88].
(4) Association between a father’s depressive moods and the child’s gender: When the child is a boy, the father’s depressive mood rises, and this change is significant (coefficient = 0.0869, standard error = 0.0304). This might be due to the societal expectations and pressures on fathers with sons. For instance, in certain cultural perspectives, boys are seen as the economic pillars of the family, possibly inducing psychological pressure on the fathers [89].

In summary, Table 13 reveals how a child’s gender impacts the psychological health of parents. The family concept of “valuing sons over daughters” and the accompanying economic pressures, such as buying a house or wedding expenses, influence parents’ psychological states to some degree. These insights provide a fresh perspective on the relationships between childbirth, familial roles, and psychological health.

Heterogeneity in effects. Contrary to the traditional view, younger parents may find it more challenging to adapt to the arrival of a newborn due to lower psychological maturity and economic stability. They may experience higher levels of anxiety and financial stress. In contrast, middle-aged and older parents often have more stable economic conditions and greater life experience, enabling them to handle the responsibilities of parenthood more effectively. Their well-established social support networks also provide additional assistance, reducing psychological stress and enhancing their parenting confidence and capabilities.Urban parents, generally equipped with better economic and educational resources, might also grapple with intensified work and life pressures. In contrast, rural parents might face significant economic challenges but could benefit from greater support from their community and extended family. Highly-educated parents might have easier access to child-rearing information and resources but might also have loftier parenting expectations. In contrast, parents with lower educational attainment might face challenges in accessing child-rearing knowledge and skills but might find more robust support within their families and communities. In certain regions, owing to traditional gender preferences (“preference for boys over girls”), the birth of a girl might impose added psychological stress on parents, affecting their happiness and mood. While both fathers and mothers are influenced by first-time childbirth, due to physiological, cultural, and societal role differences, mothers often bear a heavier burden, especially when the child is a girl.Differences between fathers and mothers. Throughout the demographic transition, while both fathers and mothers feel the impacts of first-time childbirth, mothers, due to physiological, cultural, and societal differences, often endure greater stress.

In summary, the demographic transition has not only profoundly influenced the structure and economy of nations and societies but has also ushered in significant changes in the lifestyles, values, and mental health of families and individuals. Approaching from the perspective of first-time childbirth, this study offers deep insights into how this transformation affects the psychological well-being of parents.

## Policy Recommendations

(1) Comprehensive Mental Health, Childbirth Education, and Physical Health Support. Health departments should provide free mental health counseling services in public hospitals and community health centers for first-time parents, with a particular focus on middle-aged and elderly mothers, rural mothers, and those with lower educational attainment, especially within the first six months postpartum. Governments must organize parenting education seminars and offer physical health recovery guidance, including postpartum rehabilitation courses and dietary advice. Additionally, high-cost health management items related to advanced-age childbearing—such as pelvic floor muscle repair and menopause health management—should be included in maternity insurance coverage to alleviate the long-term economic burden on middle-aged and rural mothers for health maintenance.
(2) Balancing Work with Childbirth, Building Confidence for the Future, and Providing Support: Labor and social security departments should collaborate with business associations to encourage all levels of enterprises to promote flexible work arrangements during pregnancy and maternity leave. This would provide new parents opportunities for remote work, part-time jobs, or freelancing. Simultaneously, necessary digital skills training should be provided to young parents and those with lower educational backgrounds. Furthermore, seminars on family planning and future development should be organized to instill confidence in new parents and help them strike a balance between work and family life.
(3) Community Resource Integration, Economic Cooperation, and Building Confidence for the Future: Local governments need to collaborate with rural and community development departments to promote economic cooperation among families during pregnancy and the first postpartum year at rural and urban community centers (such as promoting collective purchasing of baby supplies and organizing group parenting courses), providing specific community support and resource sharing for new mothers, especially for rural families and new mothers with low educational attainment. The government should recruit qualified parenting instructors locally to provide rural mothers and those with low educational attainment with twice-weekly in-home guidance services. These services will assist in creating parenting schedules, interpreting medical documents, and other related tasks, thereby reducing parenting anxiety stemming from knowledge gaps.
(4) Public Advocacy, Social Security, and Building Confidence for the Future: Given the identified spillover effects of first-time childbirth within village communities, cultural and publicity departments should collaborate with public media. Throughout the year, especially during peak pregnancy and childbirth periods, public advocacy campaigns should be launched across major public media platforms and community broadcast televisions. The objective of these campaigns should be to encourage the public to discard traditional preferences for boys over girls and provide all new parents with social security policy consultations. Building on this, public lectures on family future planning should be organized to encourage new parents to adopt an optimistic outlook towards the future.
